# Settling into an Increasingly Hostile World: The Rapidly Closing “Recruitment Window” for Corals

**DOI:** 10.1371/journal.pone.0028681

**Published:** 2011-12-14

**Authors:** Suzanne N. Arnold, Robert S. Steneck

**Affiliations:** Darling Marine Center, University of Maine, Walpole, Maine, United States of America; Victoria University Wellington, New Zealand

## Abstract

Free space is necessary for larval recruitment in all marine benthic communities. Settling corals, with limited energy to invest in competitive interactions, are particularly vulnerable during settlement into well-developed coral reef communities. This situation may be exacerbated for corals settling into coral-depauperate reefs where succession in nursery microhabitats moves rapidly toward heterotrophic organisms inhospitable to settling corals. To study effects of benthic organisms (at millimeter to centimeter scales) on newly settled corals and their survivorship we deployed terra-cotta coral settlement plates at 10 m depth on the Mesoamerican Barrier Reef in Belize and monitored them for 38 mo. During the second and third years, annual recruitment rates declined by over 50% from the previous year. Invertebrate crusts (primarily sponges) were absent at the start of the experiment but increased in abundance annually from 39, 60, to 73% of the plate undersides by year three. Subsequently, substrates hospitable to coral recruitment, including crustose coralline algae, biofilmed terra-cotta and polychaete tubes, declined. With succession, substrates upon which spat settled shifted toward organisms inimical to survivorship. Over 50% of spat mortality was due to overgrowth by sponges alone. This result suggests that when a disturbance creates primary substrate a “recruitment window” for settling corals exists from approximately 9 to 14 mo following the disturbance. During the window, early-succession, facilitating species are most abundant. The window closes as organisms hostile to coral settlement and survivorship overgrow nursery microhabitats.

## Introduction

Larval recruitment is one critical process leading to the recovery of community structure following disturbances in the marine realm. Yet, Caribbean coral reefs are suffering from increasing rates of disturbances and decreasing rates of recruitment [1], [2]. Myriad stressors and bottlenecks prevent passage of a coral larva through sequential life history stages to successful recruitment [3].

Free space is necessary for settling larvae, and thus, is important in all marine benthic communities [4]. Therefore, the dynamics of processes that create and colonize free space on a coral reef are critical to the successful recruitment of corals. Following perturbations, a succession of benthic organisms colonize newly bared substrate on reefs. A progression of body plans begins with solitary and vine-like forms and ends with nearly complete coverage of colonial sheet and encrusting morphologies [5]. While the slow or nonexistent rates of coral recovery from disturbance on Caribbean reefs are well documented [1], very little is known about how rates of succession affect the recruitment of reef corals.

In shallow reef environments, corals recruit to nursery microhabitats where rates of predation and competition are lower than on exposed surfaces and post-settlement survivorship is higher [6], [7]. On reefs, these nursery microhabitats can be nooks and crannies. In recruitment studies, nursery microhabitats often are the underside edge of terra-cotta settlement plates [8]. In a previous recruitment study in Bonaire, Netherlands Antilles, we found 83% of all coral recruits in the 1.5-cm outer perimeter of the plate underside, an area we termed the “subcryptic settlement microhabitat” [9]. Corals that settle in this area are out of harms way from grazing fish, yet with modest growth the developing coral colony will be in full sun and high growth conditions. It is in these microhabitats where demographically important ecological interactions are most critical, and with small reservoirs of energy to invest in competitive interactions newly settled corals are particularly vulnerable when faced with a well-developed benthic community structure [10]. Mechanisms that determine growth and mortality rates of juvenile corals, however, are only recently being investigated.

Since the 1970s, many Caribbean coral reefs have shifted from coral to macroalgal-dominated systems [11], [12], [13], and algae, even modest amounts of turf algae [9], negatively affect the recruitment of corals [14]. In our previous research in Bonaire, on a relatively healthy Caribbean reef with high coral and low macroalgal cover [15], invertebrate crusts such as sponges, bryozoans, and ascidians were absent from coral nursery habitats at the start of a recruitment study, but they steadily increased during the experiment and accounted for approximately 50% of the substrate two years later [9]. Consistent with other studies, these cryptic heterotrophs readily overgrew small corals [16]. Sponge extracts physiologically stress even adult corals on Caribbean and Pacific reefs [17]. With the rising abundance of sponges and other invertebrate crusts comes the decline in crustose coralline algae (CCA). Crustose coralline algal species are thin and recruit early [18] to the undersides of settlement plates. In Bonaire, we observed *Titanoderma prototypum*, a preferred settlement substratum for certain species of corals [19], [20], rapidly recruit and reach peak abundance (12% cover of the entire plate underside) within 5 mo of deployment of settlement plates. Other CCA reached peak abundance within 8 mo before being overgrown by invertebrate crusts. Thus, it is possible that following a disturbance that bares primary substrate, a ‘recruitment window’ exists. During this window, early succession facilitating species, such as the coralline alga *T. prototypum*, are present and inhibitory organisms, such as sponges and bryozoans, have yet to colonize nursery microhabitats for corals. While this window is maximally open, newly settled corals may be more likely to successfully “run the gauntlet” to recruitment [9].

The fate of coral larvae settling into an increasingly hostile world is addressed in this study. We sought to observe the succession of benthic organisms and their interactions in coral nursery microhabitats with newly settled corals on a typical, contemporary, macroalgae-dominated Caribbean reef. Is there an identifiable period following a perturbation on a reef when substrate conditions are optimal for successful coral recruitment? The emerging view regarding recruitment patterns is that benthic interactions, such as competition, drive patterns when space is limiting, but larval supply or very early post-settlement mortality drives spatial variability in recruitment when free space is abundant [21]. Given that any bare substrate is rapidly colonized by microbial films, diminutive turf algae, cyanobacteria, and macroalgae, is free space ever abundant and persistent on Caribbean reefs today?

We studied recruitment and survivorship of corals over three years as succession altered the species composition of nursery microhabitats. By observing interactions between corals and other sessile organisms on coral settlement plates in Belize, we sought to provide new insights into the important ecological interactions that occur within typical epibenthic Caribbean reef communities.

## Materials and Methods

### Study Area

To assess patterns in coral recruitment and early post-settlement survivorship in relation to other benthic organisms, we conducted studies on the Mesoamerican Barrier Reef in Belize (16°48.18 N 88°04.93 W) from June 2005 to August 2008. We selected two sites on the forereef adjacent to Carrie Bow Cay and two sites on the forereef adjacent to South Water Cay, just to the north. Sites were all at 10 m water depth and separated by approximately 0.5 km. The coordinates for the individual sites are as follows: Site 1- 16°48.098 N 88°04.725 W, Site 2- 16°48.363 N 88°04.472 W, Site 3- 16°48.769 N 88°04.686 W, and Site 4- 16°49.168 N 88°04.677 W.

### Site Characteristics

At each site we established 5 10-m line transects along which we addressed benthic cover. Using the line intercept transect (LIT) method [22], we stretched a 10-m tape measure just above the reef and at each centimeter identified each centimeter of substrate (live coral species, crustose coralline algae (CCA), non-coralline crusts (primarily *Peyssonnelia* spp.), macroalgae (primarily *Dictyota* spp.), articulated algae (primarily *Halimeda* spp.), turf algae, sponges/gorgonians, and sand) falling directly under the tape.

### Coral Recruitment and Early Survivorship

To study the effect of benthic organisms (at millimeter to centimeter scales) on coral recruitment and survivorship, we used 10 cm×10 cm×1 cm terra-cotta settlement plates to mimic primary substrate on a reef. We define ‘recruitment’ in an operational sense, referring to those newly settled corals that have survived metamorphosis and have recognizable skeletons, dead or alive, at the time of the retrieval of the plates. In June 2005, we deployed 25 tiles at each of the 4 sites. Five plates per transect were affixed to dead coral and separated from the bottom by a 1-cm PVC spacer. Plate deployment was modified from Mundy [23] as per Arnold et al. [9]. To the greatest extent possible, plates were placed on flat substrate to reduce the variance in the gap between the reef and the plate.

Plates were monitored in August 2005, March 2006, August 2006, August 2007 and August 2008 for newly recruited corals, survivorship of previously identified corals and succession of benthic organisms. One full day was required to sample each site. Thus the monitoring occurred over four days. In the results, we refer to the monitoring periods mentioned above as “approximate days following deployment”, or day 64 (for August 2005), day 271 (for March 2006), day 427 (for August 2006), day 802 (August 2007), and day 1157 (for August 2008). At each of the 5 monitoring intervals, all plates were transported from the reef to the lab in seawater, analyzed under a dissecting microscope (25×) while immersed in seawater, and returned to the reef within 6 h. In the Caribbean at 10 m depth, corals settle primarily on undersides of settlement plates [9], [10], [24], whereas with increasing depth and decreasing light, coral settlement shifts to upper surfaces [6]. Thus, we tracked recruitment and succession on the plate undersides only.

Optimally, when identifying coral recruits, the tissue is removed, often with bleach, to reveal the skeletal elements helpful for identification [25], [26]. Destructive sampling in this manner was not possible in this study because we sought to track survivorship of the recruits. Fortunately, identification of newly settled recruits to genus in the Caribbean is less challenging than in the IndoPacific due to lower diversity of coral assemblages. Through characteristics of the skeletal structure, accurate identification to the genus level is possible, particularly for the most abundant *Agaricia* spp. and *Porites* spp. Shearer and Coffroth [27] genetically confirmed the identity of visually labelled *Agaricia* spp. and *Porites* spp. recruits as *Agaricia agaricites* (or *Agaricia tenuifolia*- the two species were indistinguishable genetically at the time of the study) and *Porites astreoides*. *Porites* spp. are characterized by a porous coenosteum, septa with prominent teeth, often an epitheca, and they can form multiple polyps within a short time (wks-mos). *Agaricia* spp. do not develop an epitheca and have prominent, laminar septa, and can remain in single polyp form for over a year. Thus, newly settled corals were identified to genus where possible, measured at maximum diameter, and mapped relative to an x-y coordinate scale around the plate. We recorded the substrate upon which the coral recruited and the percent cover of all other encrusting biota on the plate underside (crustose coralline algae, non-coralline algal crusts, macroalgae, articulated algae, turf algae, sponges and bryozoans and polychaete worm tubes). To determine percent cover, we used visual estimates because this method was shown to be more accurate and have less within-observer variation than random-point-quadrats (RPQ), particularly when estimating cover of rare sessile organisms [28].

While plates were searched several times at each observation period for coral recruits, carefully looking under foliose algae, it is possible that recruitment rates would have been higher if plates were bleached and dried before searching. This methodological difference should be taken into consideration if recruitment rates reported in this study are compared to other recruitment studies in the region.

### Substrate Preference and Availability

Special attention was paid to the “subcryptic settlement microhabitat” [9], or the outside 1.5 cm edge of the plate underside where 89% of corals settled. Hereafter, we will refer to this area as simply the “nursery microhabitat”. For each nursery microhabitat, we mapped the substrate cover of all identifiable biota and recorded substrates on which newly recruited corals first appeared. We then plotted the percent of coral spat occupying each substrate as a function of that substrate's abundance. In this way, spat settling in proportion to the abundance of biotic substrate in that habitat on a particular plate are neither preferentially selecting nor avoiding any substrate. Spat found at higher proportion than substrate availability are either selectively choosing, or surviving better, on that substrate. We interpret those substrates to be recruitment facilitators. Conversely, spat found at lower proportions than substrate availability are either avoiding, or dying on, that substrate. To compare substrate selection, for each substrate we calculated a selection ratio w_i_, which is the proportional use divided by the proportional availability of each substrate.

### Analyses and Data Treatment

To test whether pooling data from the four sites was justified, we first used non-metric multidimensional scaling ordination (MDS, using PRIMER v6 [29]) on percentages of benthic cover from each of the five 10-m transects at the four sites to determine among-site variance. One-way analysis of similarity (ANOSIM) was used to analyze the importance of site on benthic cover that can contribute to larval supply (adult live coral cover) and recruitment potential (abundance of CCA, macroalgae, articulated algae, turf algae, and sponges). We then calculated the mean recruit density per settlement plate per site (n = 25 per site) and performed a one way ANOVA on non-transformed data. We used these pooled data for all of our analyses. Thus, substrate composition analyses are performed on 100 plates. Due to the high proportion of recruits settling onto the outside underside edge of the plates (222 out of 249 recruits), most analyses include only the outside 1.5 cm edge of the plate underside. These analyses are identified as such and refer to this 1.5 cm area as the ‘subcryptic settlement microhabitat’ or simply ‘nursery microhabitat’.

## Results

### Biotic Characteristics and Recruitment Among Sites

The surrounding biota that might influence coral recruitment varied little among sites (non-metric multidimensional scaling MDS). Site weakly influenced adult live coral cover, abundance of CCA, macroalgae, articulated algae, turf algae, and sponges (one-way analysis of similarity ANOSIM, Global R = 0.11, *P* = 0.06). Furthermore, there was no significant difference in total recruitment among sites (one-way ANOVA, *P* = 0.89). At the end of the 3.17-yr experiment, mean coral recruits (± SE) per entire plate underside were: Site 1 = 2.43±0.09, Site 2 = 2.59±0.10, Site 3 = 2.79±0.14, Site 4 = 3.0±0.26. Thus, we pooled recruitment data from the four sites for all subsequent analyses.

### Temporal Trends of Coral Recruitment

**Figure 1:**
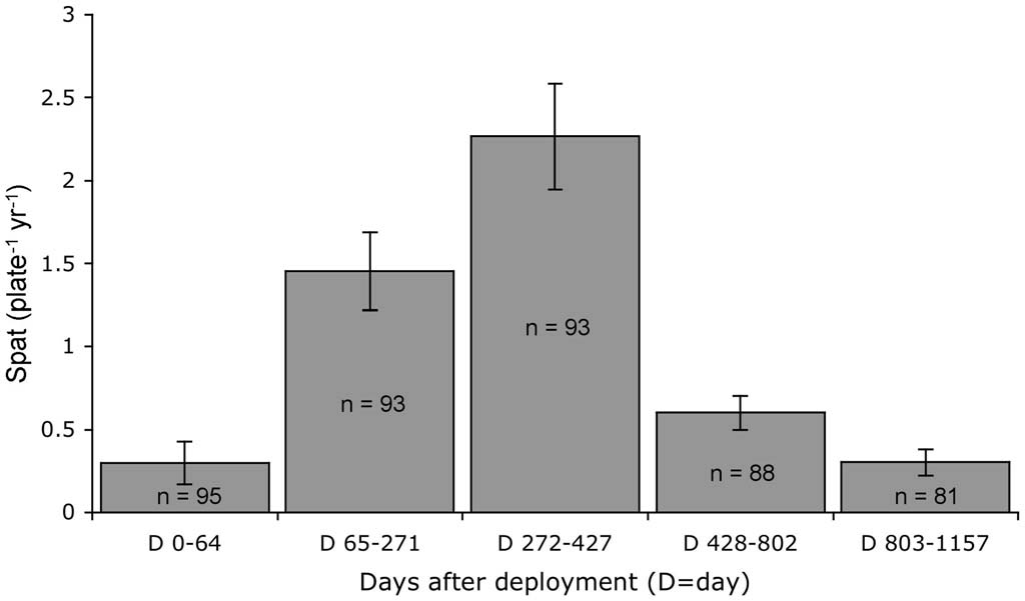
Rates of coral recruitment. Mean number of spat per plate underside during the five sampling intervals ± SE were 0.30±0.13, 1.45±0.23, 2.26±0.32, 0.60±0.10, 0.30±0.08. Error bars are ± SE.

Rates of coral recruitment peaked after year one and steadily declined during years two and three by over 50% from the previous year (Fig. 1). *Agaricia* was the predominant genus identified, making up 74% of all spat. Twenty-four percent were *Porites* spp, with the remaining 2% unidentified. Eighty-nine percent of all spat settled in the 1.5-cm outside edge of the plate underside. For this reason, all further analyses applied to species composition and settlement in this nursery microhabitat.

### Settlement Preference and Substrate Composition

**Figure 2:**
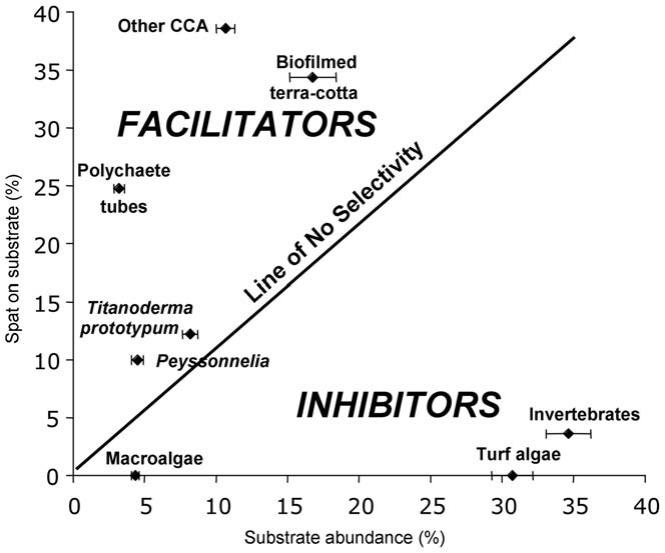
Proportional recruitment success per occupant of nursery microhabitats. Selectivity of recruits shown by the percent cover of settlement substrates growing on the 1.5-cm perimeter of plate undersides, with corresponding recruitment and a diagonal line of no selectivity. Selectivity data were based on 223 newly settled spat in this area. Separate analyses for each substrate included only the subset of plates on which the substrate was present. The y-axis represents the number of spat on each substrate as a percentage of the total number of recruits on that subset of plates. Percent cover of fouling organisms on the plate underside was recorded at the time of first observation of the newly settled spat.

Selection ratios (w_i_) of all substrates, ranging from high to low, were polychaete tubes = 8.30, crustose coralline algae (not including *T. prototypum*) = 3.45, *T. prototypum* = 2.50, biofilmed terra-cotta = 1.90, *Peyssonnelia* = 1.40, invertebrate crusts = 0.11, turf algae = 0.03, and macroalgae = 0.00. A w_i_ value greater than 1 indicates a positive selection for the substrate and a value less than 1 indicates avoidance of the substrate. A value near 1 indicates that the resource was used proportionally to its availability and no resource selection was observed. Thus, we deduce coral recruitment success to vary along the spectrum of substrates ranging from those that facilitate to those that inhibit this process (Fig. 2).

**Figure 3:**
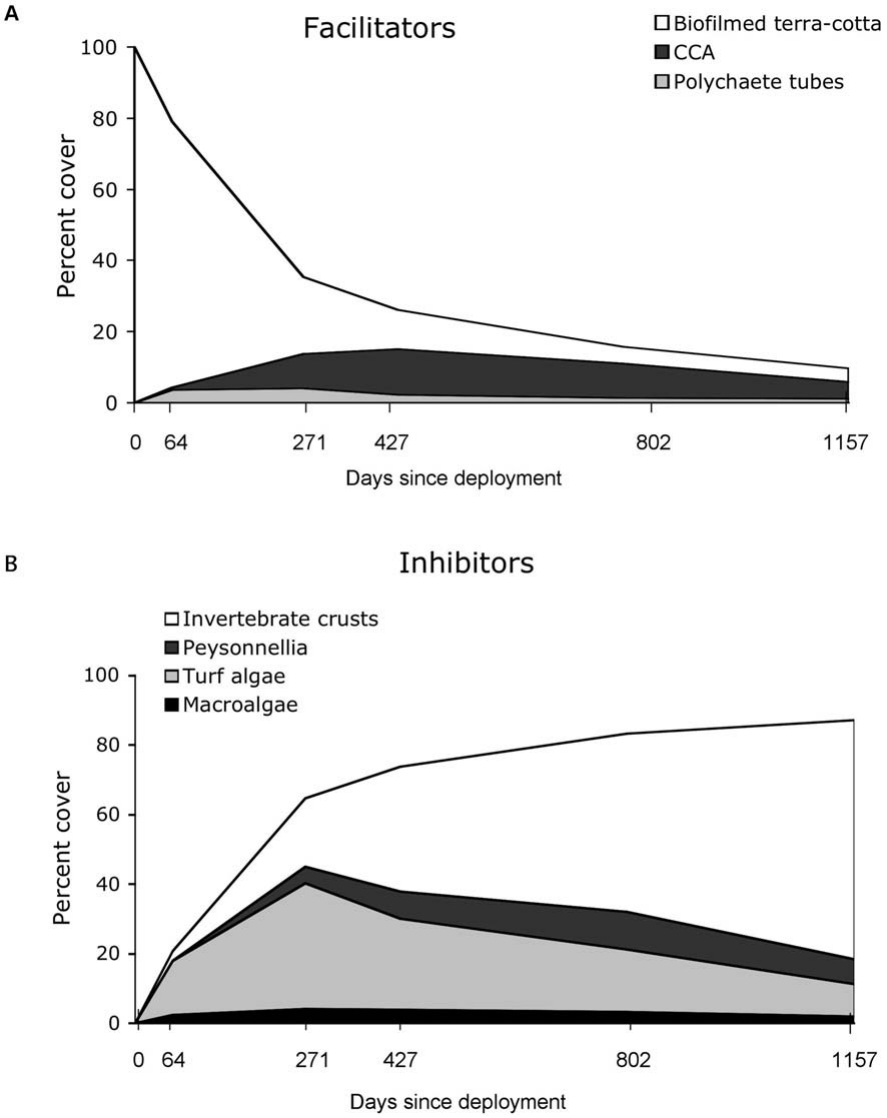
Substrate composition over time on the plate nursery microhabitats. Succession of recruitment **A** facilitators and **B** inhibitors.

Coral spat settled preferentially on early successional substrates, such as biofilms, polychaete tubes, and coralline algae, which declined over time in the subcryptic nursery habitat (Fig. 3A). Abundance of these recruitment facilitators peaked at approximately one year, with polychaete tubes reaching a peak abundance of 4% at day 271 and CCA reaching 13% at day 427. The biofilmed terra-cotta, or “free” space, declined rapidly, making up only 22% of the subcryptic settlement habitat by day 271 (Fig. 3A). Conversely, plates increasingly fouled with species inimical to settlement and survival, i.e., recruitment inhibitors (Fig. 3B). Turf algae and to a lesser extent macroalgae peaked at day 271, whereas *Peyssonnelia* spp. peaked at 11% abundance at day 802, and heterotrophic invertebrate crusts (primarily sponges) continued to increase in abundance through day 1157, peaking at 69%. By the end of the study (day 1157), all inhibitor species combined accounted for 87% of the nursery microhabitat (Fig. 3B).

**Figure 4:**
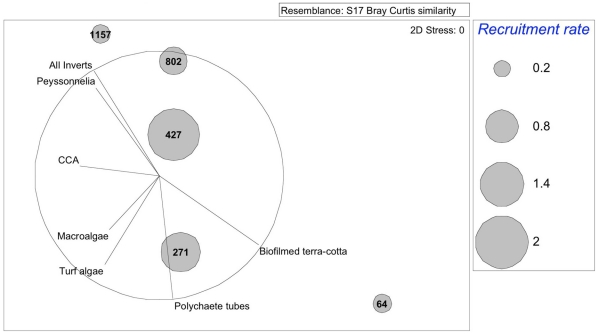
Substrate composition over time and corresponding recruitment rates. Dissimilarity of substrate composition at each sampling interval (day 64, 271, 427, and 1157) with the number of new recruits per plate per 365 d. Number of days deployed had a strong influence on substrate composition (analysis of similarity ANOSIM, R = 0.36, *P* = 0.001). Substrate categories are given as vectors indicating dominant substrates. Invertebrate crusts accounted for 42% of the total dissimilarity between day 64 and day 1157 (similarity percentage analysis).

Changes in substrate composition of nursery microhabitats were examined (non-metric multidimensional scaling ordination, MDS; Fig. 4). The relative importance of immersion time (64, 271, 427, 802, 1157 d) and site on community composition was evaluated using two-way ANOSIM based on the Bray-Curtis Similarity Coefficient [30]. We found that site had an extremely weak influence (R = 0.049, *P* = 0.002), whereas days deployed had a strong influence (R = 0.36, *P* = 0.001). Similarity percentage (SIMPER) analysis [30] revealed that the average dissimilarity between the community structure of the plate undersides at the various sampling intervals was 60.26%, with the highest dissimilarity, logically, between day 271 and 1157 (0.75), with encrusting invertebrates causing 42% of total dissimilarity between the initial and final sampling interval. Corresponding mean rates of coral recruitment, in the nursery microhabitat during each interval, peaked around year one (Fig. 4), and vectors of particular substrates overlaid on the MDS ordination illustrate the shift to invertebrate crusts as succession progresses.

**Figure 5:**
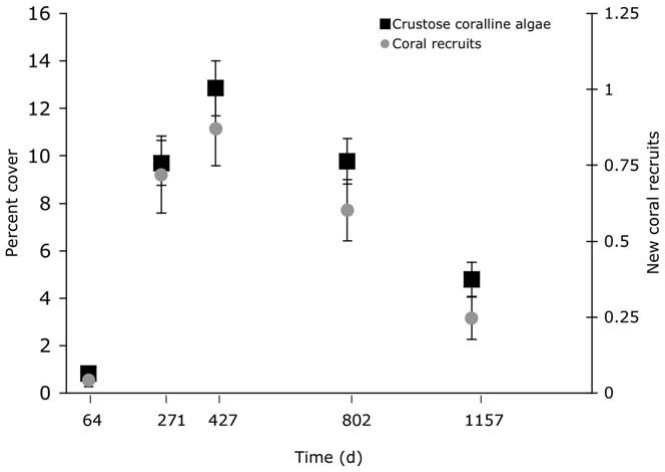
Coral recruits and crustose coralline algae over time. The number of new coral recruits censused at each monitoring period in the 1.5-cm outer edge of the plate underside and the corresponding cover of all CCA species (*Titanoderma prototypum* and all other CCA) in that area.

Notably, the number of new recruits per monitoring period tracked the abundance of crustose coralline algae in the nursery microhabitat (Fig. 5). Both the number of new recruits and the abundance of CCA peaked just after one year and then steadily declined.

**Figure 6:**
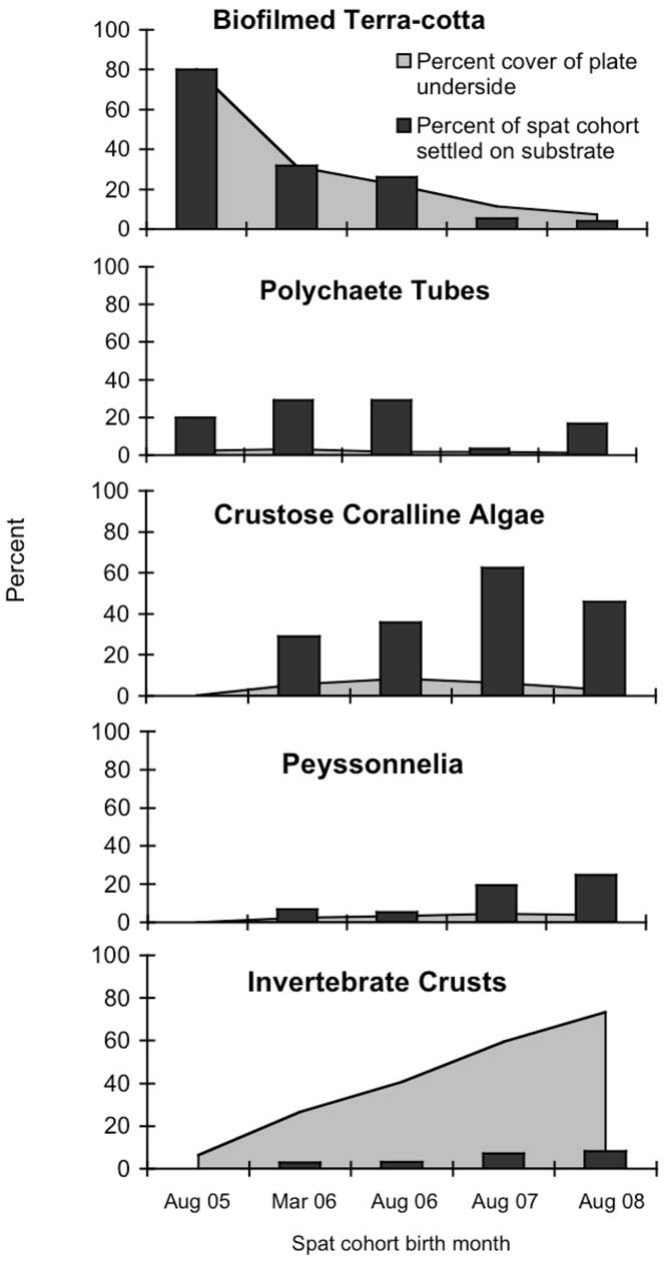
Spat settlement choice relative to substrate abundance over time on plate undersides. Bars show percentage of spat from each observed cohort (labeled on the x-axis) recruited to the substrate. Substrate percent cover shown in light gray over time.

Settlement substrate choice changed over the course of the study as settling coral larvae faced a more diverse and developed benthic community. Earlier-recruiting cohorts chose the biofilmed terra-cotta, worm tubes, *Titanoderma prototypum* and other CCAs, whereas, later spat recruited more onto increasingly abundant peyssonnelids and invertebrate crusts (mostly bryozoans, but in a few cases sponges) (Fig. 6). These latter substrates were likely poor choices for newly settled corals. Sponges accounted for over half of all recorded spat mortality, followed by peyssonnelids (Table 1).

**Table 1:** Autopsy report.

Killer	Number of spat killed	Percentage of dead
Sponge	99	50.3
*Peyssonnelia* spp.	14	7.1
Bryozoan	8	4.1
Turf algae	6	3.0
CCA	4	2.0
*Dictyota* spp.	3	1.5
*Lobophora* sp.	3	1.5
Gypsina	2	1.0
Undetermined	55	27.9

Total number and percentage of spat overgrown by substrate.

### Early Survivorship

**Figure 7:**
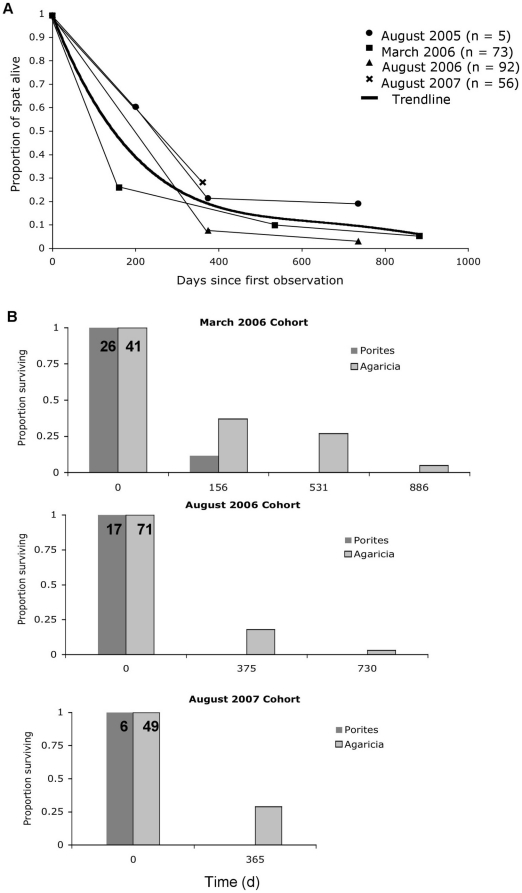
Survivorship curves by cohort and species. **A** Proportion of spat alive at each subsequent monitoring period. A fourth-order polynomial curve fits all cohorts (in bold), highlighting an average survivorship of ∼20% after 365 d and overall mortality converging to >90% by the end of the study. **B** Survival of three cohorts of *Porites* spp. and *Agaricia* spp. over time.

No increased survivorship of earlier cohorts was obvious (Fig. 7A). For example, the last cohort (August 2007) had higher survivorship than previous cohorts. Despite the slightly different trajectories, average survivorship was just under 10% by the end of the study (Fig. 7A).

**Figure 8:**
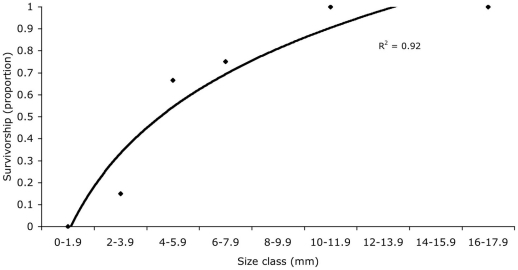
Probability of survival of August 2007 *Agaricia* spp. cohort after one year by size class (2-mm diam. size classes after one yr; n = 49 *Agaricia* spp).

Differences in survivorship between the two genera, *Agaricia* and *Porites* revealed *Porites* spp.'s inability to compete with the increasingly hostile environment, with no survivorship beyond the first cohort (Fig. 7B). As corals grow, their survivorship increases (Fig. 8). Larger individuals of *Agaricia* spp. had an increased likelihood of surviving the first year even in the well developed, and largely hostile, community structure. Those individuals ≥10 mm in diameter had 100% survival over the next year, whereas recruits ≤2 mm had zero survival (Fig. 8).

## Discussion

Receptivity of nursery microhabitats to settling corals peaked after approximately one year (Fig. 1). This timing suggests a ‘recruitment window’ exists, or a period in this case about 9 to 14 mo. after a space has opened on a reef, during which the benthic composition is optimal for coral recruitment. Corals were twice as likely to recruit to settlement plates in year one than in year two and three times more likely than in year three (Fig. 1).

Spat settled preferentially on biofilms, calcareous polychaete tubes, and crustose coralline algae, suggesting demographic importance of facilitator substrates (Fig. 2). These substrates were all relatively early successional stages (Fig. 3A), with organisms of later successional stages inimical to settlement and survivorship rising in dominance over time (Fig. 3B). Thus, a dynamic balance between the positive effects of recruitment facilitators and the negative effects of recruitment inhibitors, particularly among encrusting sponges, may limit both coral recruitment and early survival (Figs. 2, 6, Table 1).

Succession in coral nursery habitats, as it affects coral recruitment, is largely unstudied. We found functional group (growth form) changes conforming to patterns described by Jackson [5]. After colonization by microbial biofilms, aclonal, solitary polychaete tubes increased in abundance, but all surfaces became dominated by encrusting morphologies of calcified (coralline) algae, non-coralline (*Peyssonnelia* spp.) algae, and invertebrates (bryozoans, ascidians, and sponges). Among encrusting marine organisms, thicker margins overgrow and thus outcompete thinner margins [31], [32]. Accordingly, thin facilitating corallines such as *Titanoderma prototypum* (crust thickness 40 mm) lose space competition to thicker, overgrowing organisms. Replacement of thin, early-succession facilitating species by thick late successional species often ends with the thickest sponges covering everything and sometimes completely filling the nursery habitat (personal observation). We have monitored one sponge-filled nursery habitat in Belize for eight years with most of the last five years remaining in a sponge-filled state.

Recruitment rates fell as the nursery microhabitat shifted towards encrusting invertebrates and away from biofilms, CCA, and polychaete tubes (Figs. 3, 5). Surprisingly, however, we recorded an increase in the proportion of recruits settling on substrates inimical to survival (Fig. 6). Thus, as the percent cover of invertebrate crusts (primarily sponges) increases, so too does the proportion of spat recruiting to these substrates. With sponges in particular accounting for over 50% of all recorded spat mortality in this study, this settlement choice seems maladaptive. However, it is unclear how much can be inferred in these instances about larval behavior. If succession on contemporary Caribbean reefs is progressing towards benthic organisms that grow faster than corals, the beneficial adaptive larval behavior of corals may become irrelevant [10], [24], [33]. A more plausible explanation is that the recruitment potential of the benthos was so reduced that settling larvae with diminished energy reservoirs had little choice but to settle on invertebrate crusts and peyssonnelids.

Regardless of selected recruitment substrate, percent survivorship of recruits over the course of the study converged to around 10% (Fig. 7A). Other studies have shown that settlement preferences were linked to reduced post-settlement mortality [34], [35], [36]. Although we observed recruitment preferences for CCA, as shown in other studies [19], [20], [33], spaces occupied by CCA (and any recruits that may have settled there) were rapidly overgrown by faster growing invertebrate crusts. For *Porites* spp. recruits, all but those in the earliest cohort (March 2006) that faced relatively hospitably fouled plates had zero survivorship, indicating that they may be highly vulnerable to overgrowth in well-developed settlement microhabitats (Fig. 7B).

For *Agaricia* spp., we found close correlation between size of recruit and survivorship (Fig. 7). The concept of a size refuge for many corals has been demonstrated by others, particularly in the IndoPacific where fast-growing species are more abundant ([16], [37], [38], and also [39] in the Caribbean). The relatively fast-growing Caribbean acroporids rarely recruit [40], [41], [42], [43], however, and many scientists report a shift in community dominance away from the major framework builders, *Acropora* and *Montastrea*, to the slow-growing coral genera *Agaricia*, *Porites*, and even a predominance of sponges [44], [45]. The long-term consequences of this shift to slower growing coral species and potentially more prolific sponge populations are unknown, but with coral growth rates being important for outcompeting encrusting invertebrates like sponges, Caribbean reefs may be particularly vulnerable.

Since “survival of the thickest” applies, take-over by encrusting invertebrates effectively halts coral recruitment and prevents subsequent coral recovery in that microhabitat. Historically, this scenario may have always been the case, but Caribbean reefs today are suffering from a loss of architectural complexity [46] caused primarily by the post-1980 decline in branching acroporids [47]. The presence of fewer branching corals likely reduces the frequency of coral fragmentation, which, combined with the algal smothering of the reef, has led to a greatly reduced number of nooks and crannies for coral settlement. Today, these spaces are most commonly colonized by diminutive species of the genera *Agaricia* and *Porites*, which thereby lead the thrust of any recovery in the Caribbean [44], [45]. It is possible that the balance has shifted on Caribbean reefs, with increasingly rare nursery microhabitats shifting rapidly to hostile encrusting organisms and closing recruitment windows quickly.

Compared to IndoPacific reefs, Caribbean reefs have distinct disadvantages when it comes to recovering following disturbances. The Caribbean has naturally lower biodiversity, particularly of the reef-building, branching corals that never readily recruited even when they were abundant [40]. Sexual recruits of Caribbean *Acropora* spp. are rare and populations are maintained primarily by fragmentation [48]. In contrast, IndoPacific reefs have weedy *Acropora* spp. that readily recruit and rapidly grow, accelerating recovery and the creation of more recruitment windows. Further, it is possible that overfishing parrotfishes could increase substrates inimical for coral recruitment [49]. Higher fleshy algal biomass, due to reduced herbivory in the Caribbean, could shade nursery microhabitats, shifting them from facilitating and neutral autotrophs (corallines and *Peyssonnelia* spp.) to inhibiting and deadly heterotrophs (sponges) [9]. If so, improved management of herbivores could slow the closure of recruitment windows on Caribbean coral reefs.
